# Plasma and Urinary Biomarkers Improve Prediction of Mortality through 1 Year in Intensive Care Patients: An Analysis from FROG-ICU

**DOI:** 10.3390/jcm12093311

**Published:** 2023-05-06

**Authors:** Beth A. Davison, Christopher Edwards, Gad Cotter, Antoine Kimmoun, Étienne Gayat, Agnieszka Latosinska, Harald Mischak, Koji Takagi, Benjamin Deniau, Adrien Picod, Alexandre Mebazaa

**Affiliations:** 1 Inserm UMR-S 942, Cardiovascular Markers in Stress Conditions (MASCOT), University of Paris, 75010 Paris, France; gadcotter@momentum-research.com (G.C.); etienne.gayat@aphp.fr (É.G.); benjdeniau@gmail.com (B.D.); adrien.picod@inserm.fr (A.P.); alexandre.mebazaa@aphp.fr (A.M.); 2 Momentum Research, Inc., Durham, NC 27713, USA; chrisedwards@momentum-research.com (C.E.); kojitakagi@momentum-research.com (K.T.); 3 Service de Médecine Intensive et Réanimation Brabois, CHRU de Nancy, Université de Lorraine, 54511 Nancy, France; akimmoun@gmail.com; 4 Inserm U1116, F-CRIN INI-CRCT, 54500 Nancy, France; 5 Department of Anesthesia, Burn and Critical Care, University Hospitals Saint-Louis—Lariboisière, AP-HP, 75010 Paris, France; 6 Université Paris Cité, 75006 Paris, France; 7 Mosaiques Diagnostics GmbH, D-30659 Hannover, Germany; latosinska@mosaiques-diagnostics.com (A.L.); mischak@mosaiques-diagnostics.com (H.M.)

**Keywords:** intensive care units, critically ill, mortality, biomarkers, severity score, prognosis

## Abstract

Background: This study aimed to assess the value of blood and urine biomarkers in addition to routine clinical variables in risk stratification of patients admitted to ICU. Methods: Multivariable prognostic models were developed in this post hoc analysis of the French and EuRopean Outcome ReGistry in Intensive Care Units study, a prospective observational study of patients admitted to ICUs. The study included 2087 patients consecutively admitted to the ICU who required invasive mechanical ventilation or a vasoactive agent for more than 24 h. The main outcome measures were in-ICU, in-hospital, and 1 year mortality. Results: Models including only SAPS II or APACHE II scores had c-indexes for in-hospital and 1 year mortality of 0.64 and 0.65, and 0.63 and 0.61, respectively. The c-indexes for a model including age and estimated glomerular filtration rate were higher at 0.69 and 0.67, respectively. Models utilizing available clinical variables increased the c-index for in-hospital and 1 year mortality to 0.80 and 0.76, respectively. The addition of biomarkers and urine proteomic markers increased c-indexes to 0.83 and 0.78. Conclusions: The commonly used scores for risk stratification in ICU patients did not perform well in this study. Models including clinical variables and biomarkers had significantly higher predictive values.

## 1. Introduction

Intensive care unit (ICU) services are now routinely provided to a wide range of populations and have had a beneficial impact on critical care mortality [1]. However, ICU mortality and one-year all-cause mortality after ICU admission remain high, ranging from 8 to 33% and 26 to 63%, respectively [2,3,4,5]. The long-term outcomes are dismal even for patients who are discharged alive [6,7,8]. Therefore, risk stratification on admission is essential in guiding clinical management and identifying high-risk patients who should be considered for further assessments and treatment. 

Traditional clinical variables such as demographic factors, etiology, comorbidities, and routine laboratory assessments have been reported to be associated with short- and long-term mortality in ICU patients [9]. These variables were formulated over the years in several candidate prognostic models. These include the Acute Physiology and Chronic Health Evaluation (APACHE) [10,11], the Simplified Acute Physiology Score (SAPS) [12], and the Sequential Organ Failure Assessment (SOFA) [13,14]. However, their main role is to simplify such risk assessment into a simple quantifiable number but their discriminative value beyond the routine clinical assessments is limited. 

In recent years, biomarkers have been increasingly used in critically ill patients as they are expected to be useful for diagnosis, risk profiling, assessment of treatment response, and prognosis in the ICU. Indeed, a recent publication from our group showed that elevations at discharge in each of the cardiac (N-terminal pro-B type natriuretic peptide [NT-proBNP] and soluble-ST2 [sST2]) and vascular (bio-active adrenomedullin [bio-ADM]) biomarkers improved the prediction of mortality risk in patients who were discharged alive from the ICU [9]. Extending the observations from the previous study, we hypothesized that the addition of novel biomarkers representing different pathophysiological pathways to traditional clinical risk prediction models would improve the ability to assess the risk of adverse outcomes in ICU patients. In the current secondary analysis, we aimed to assess the added value of biomarkers to conventional clinical variables in predicting risks of short- and long-term outcomes in ICU patients, using data from the French and EuRopean Outcome ReGistry in Intensive Care Units (FROG-ICU) study [2,9].

## 2. Materials and Methods

### 2.1. Data Source

FROG-ICU was a prospective multicenter cohort study that included 2087 consecutive patients admitted to 21 ICUs in France and Belgium between August 2011 and June 2013 (NCT01367093) [2]. The study was approved by our Institutional Review Board (IRB) (Comité de Protection des Personnes—Ile de France IV, IRB n°00003835 and Commission d’éthique biomédicale hospitalo-facultaire de l’hôpital de Louvain, IRB n°B403201213352). Patients requiring invasive mechanical ventilation or a vasoactive agent for more than 24 h, aged ≥18 years, with social security coverage, and without severe head injury, brain death, or persistent vegetative state, not pregnant or breastfeeding, without a transplant in the prior 12 months, and not moribund were eligible. The study was approved by applicable ethics committees. The patients’ written consent was waived by ethics committees and the patients’ or next of kins’ oral consent was documented by the investigator. ICU survivors were followed for 1 year following discharge using telephone calls and postal questionnaires at 3, 6, and 12 months. A total of 2250 patients were to be included in the study [9]. Potential predictors for the models included clinical data collected at the time of ICU admission including demographics, chronic treatments, co-morbidities, and ICU admission diagnosis, as well as composite scores. Clinical signs and local laboratory measures at study inclusion and medications administered between admission and study inclusion were considered, along with biomarkers measured centrally from plasma and urine samples taken at study inclusion. Estimated glomerular filtration rate (eGFR) was derived from creatinine at inclusion using the simplified Modification of Diet in Renal Disease Study (sMDRD) equation [15]. The APACHE II score was derived from admission history and signs and lab values at inclusion [10]. Variables with >30% missing values were excluded. Where both central and local laboratory measurements were available, in most cases, the central lab measurement was used because the data were more complete. To address potential multicollinearity issues, correlations between all continuous variables were assessed. Mean blood pressure was excluded because it is a linear combination of diastolic and systolic blood pressure, and creatinine because it was highly correlated with eGFR. Among highly correlated biomarkers, NT-proBNP was chosen over BNP and troponin T over troponin I based on higher chi-square statistics and lower Akaike Information Criterion (AIC) values for univariable associations with in-ICU mortality. Values outside clinically plausible ranges were set to missing for analysis. Log-transformations were considered for highly skewed variables, particularly biomarker measures. Descriptive statistics of the full set of potential continuous and categorical candidate predictors are given in the Supplementary Material online: Table S1a,b, respectively. Blood samples (in EDTA and aprotinin) and urine samples were collected at study inclusion and biomarkers were measured centrally. Supplementary Material online: Table S2 provides detail regarding central biomarker assays.

### 2.2. Outcomes

The outcomes of interest included in-ICU, in-hospital, and 1 year mortality from the time of study inclusion. The 1 year survival time for patients who did not die was censored at the earlier of 365 days after inclusion or the individual end of study date.

### 2.3. Model Development

In-ICU and in-hospital mortality were analyzed using logistic regression. Time to 1 year mortality was analyzed using Cox proportional hazards models. To account for missing values, 10 imputed datasets were generated using the R “mice” package [16]. The imputation model included both the outcome and all potential candidate predictors. The significance of the non-linear component of a restricted cubic spline transformation was tested and, where significant, a quadratic, cubic, log2, or linear spline transformation was selected based on the AIC value and inspection of the plot of predicted outcome probability by predictor value. Fast backward selection utilizing the R package “rms” was used to determine the predictors in the final model with a p-value criterion of 0.05 for staying in the model [17]. Estimates and associated standard errors for the predictors were combined across the 10 imputed datasets using Rubin’s algorithm. Prognostic models were developed incrementally in the following manner:

- Model 1 (Clinical): An initial model was developed considering standard clinical parameters including demographics, cardiovascular and non-cardiovascular co-morbidities, admission diagnosis, and conventional lab results measured either locally or centrally.
- Model 2 (Clinical + scores): Scores including SOFA, SAPS II, Glasgow Coma Scale (GCS), Charlson Comorbidity Index, APACHE II, and Kidney Disease Improving Global Outcomes Acute Kidney Injury (KDIGO AKI) Staging Score were added to final Model 1 and backward selection, forcing final Model 1 predictors to stay in the model, was run.
- Model 3 (Clinical + scores + biomarkers): Plasma and urine biomarkers, as well as biomarkers derived from a urinary proteomic panel (HF1, HF2, CAD238, CKD273, and ACM128) [18,19,20,21,22], were added to final Model 2 and backwards selection, forcing final Model 2 predictors to stay in the model, was run.
- Model 4 (Clinical + scores + biomarkers + treatments): Chronic treatments and medications administered between admission and study inclusion were added to those predictors included in final Model 3 and backwards selection, forcing the final Model 3 predictors to stay in the model, was run.

We assessed three additional models which included as predictors SAPS II score alone, APACHE II score alone, and age and eGFR. Unadjusted and adjusted associations of the predictors with in-ICU and in-hospital mortality are presented as odds ratios (ORs) with 95% confidence intervals (CIs). Hazard ratios (HRs) with 95% CIs are given for 1 year mortality. ORs and HRs are presented for the 75th versus 25th percentiles for predictors where a non-linear transformation was used. Effects for a doubling of the value are presented for log2-transformed variables. 

### 2.4. Model Diagnostics

The c-index, pooled across the 10 imputed datasets, was computed for each of the final models as a measure of discrimination. The difference and associated 95% CIs in the c-index between the models was derived using 100 bootstrap samples. The Nagelkerke R2 index, pooled across the imputed datasets, was also computed as a measure of model performance. Each of the final models’ discrimination and calibration is further described through receiver operating characteristic (ROC) curves and calibration plots using the first imputation dataset. The likelihood-ratio test was used to compare the goodness-of-fit between each nested model. 

SAS version 9.4 (SAS Institute, Cary, NC, USA) and R version 3.5.1 software [23] was used for all analyses.

## 3. Results

### 3.1. Participants

A total of 2087 patients were included in the FROG-ICU cohort. The in-ICU and in-hospital mortality outcomes were available for all 2087 patients; 69 patients with incomplete follow-up were censored for the 1 year mortality outcome. The median (25th, 75th percentile) time from admission to inclusion was 4.0 (3.0, 6.0) days. The median follow-up from admission was 367 days. Most (86.3%) of the patients were missing at least one candidate predictor. 

### 3.2. Outcomes

**Figure 1:**
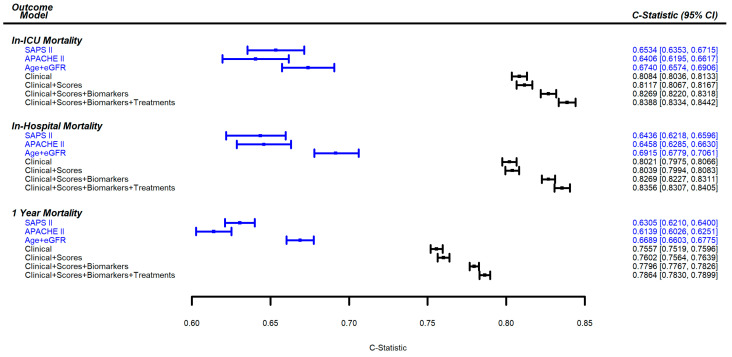
C-index values with 95% confidence intervals for incremental multivariable models for in-ICU mortality, in-hospital mortality, and 1 year mortality. In blue, additional models that include only the specified covariates.

For all outcomes, the unadjusted and multivariable-adjusted associations estimated from the incremental models did not differ much. The c-index values for final models 1–4 and the additional 3 models are shown for each outcome in Figure 1. A calibration plot for final model 4, and the preceding nested models, is presented for each of the outcomes in the Supplementary Material online: Figure S1. The corresponding ROC curves for these models are presented in the Supplementary Material online: Figure S2.

### 3.3. In-ICU Mortality

A total of 452 (21.7%) patients died during the ICU stay. The parameter estimates for the final model 4, as well as the performance characteristics of the preceding nested models, are given in Table 1. In model 1 (clinical variables), higher age, cardiac reason for ICU admission, lower diuresis of 24 h, and higher lactate were strongly associated with an increased risk of the outcome. This initial model had a c-index of 0.8084 and a Nagelkerke R2 of 0.3110. In model 2 (clinical + scores), only SAPS II score remained, with an increase in c-index of 0.0033 (95% CI 0.0023–0.0043) compared to model 1. Among the biomarkers that remained in the final model 3 after backwards selection, higher levels of interleukin 6 (IL-6), sST2, and procalcitonin (PCT) along with the urinary proteomic markers CKD273 and HF1 were shown to be associated with the outcome. This model had a c-index of 0.8269—an increase over model 2 of 0.0152 (95% CI 0.0163–0.0207)—and a Nagelkerke R2 of 0.3564. In model 4 (clinical + scores + biomarkers + treatments), treatment with aldosterone antagonists, the use of parenteral or enteral nutrition, and a cardiac arrest prior to inclusion, were associated with a higher risk of outcome. Final model 4 had a Nagelkerke R2 of 0.3707 and a c-index of 0.8388 and an increase of 0.0119 (95% 0.0095–0.0142) compared to model 3. The results of the likelihood-ratio tests showed that the additional covariates improved the overall model fit at each stage (Table 1). The models that included only SAPS II, only APACHE II score, and only age and eGFR, had c-index values of 0.6534, 0.6406, and 0.6740, respectively.

### 3.4. In-Hospital Mortality

A total of 575 (27.6%) patients died during the initial hospitalization. The parameter estimates for the final model 4, as well as performance characteristics of the preceding nested models, are given in Table 2. In model 1 (clinical), higher age, cardiac reason for ICU admission, and lower diuresis were strongly associated with a higher risk of outcome. This model’s c-index was 0.8021 and Nagelkerke R2 was 0.3097. In model 2 (clinical + scores), only SAPS II score remained after backwards selection, with an increase in c-index of 0.0018 (95% CI 0.0013–0.0024) compared to model 1. Among the biomarkers that remained in final model 3 after backwards selection, higher levels of bio-ADM, IL-6, galectin-3, PCT, and sST2 and the urinary proteomic biomarker HF1, were associated with the outcome. This model’s c-index was 0.8269, an increase over model 2 of 0.0230 (95% CI 0.0214–0.0246). The Nagelkerke R2 was 0.3605. In model 4 (clinical + scores + biomarkers + treatments), the use of parenteral nutrition and cardiac arrest prior to inclusion were associated with a higher risk of outcome. The use of morphine and antidiabetics were associated with a lower risk. Final model 4′s Nagelkerke R2 was 0.3651 and c-index was 0.8356, an increase of 0.0087 (95% CI 0.0067–0.0107) compared to model 3. The results of the likelihood-ratio tests showed that the addition of covariates improved model fit at each step (Table 2). The models that included only SAPS II, only APACHE II score, and only age plus eGFR, had c-index values of 0.6436, 0.6458, and 0.6915, respectively.

### 3.5. One Year Mortality

A total of 768 (36.8%) patients died within 1 year of study inclusion. The parameter estimates for final model 4, as well as performance characteristics of the preceding nested models, are given in Table 3. In model 1, higher age, cardiac reason for ICU admission, history of chronic liver disease, active recent malignant tumors, and loss of autonomy, were highly associated with the outcome. The model’s c-index was 0.7557 and the Nagelkerke R2 was 0.2584. In model 2 (clinical + scores), after adjusting for those parameters from model 1, a higher Charlson comorbidity index and higher SAPS II score were shown to be associated with the outcome. The inclusion of these scores increased the c-index by 0.0044 (95% CI 0.0029-0.0059) compared to model 1. This model had a Nagelkerke R2 of 0.2617. Among the biomarkers that remained in final model 3 after backwards selection, higher levels of bio-ADM, galectin-3, sST2, IL-6, PCT, and the urinary proteomic markers HF1 and ACM128, were significantly associated with the outcome. This model’s c-index was 0.7796, a significant increase of 0.0195 (95% CI 0.0168–0.0222) compared to Model 2. The Nagelkerke R2 was 0.3187. In model 4 (clinical + scores + biomarkers + treatments), chronic use of aldosterone antagonists, morphine, and nitrates, use of parenteral nutrition, renal replacement therapy, and morphine between admission and study inclusion, remained in the model. Cardiac arrest prior to inclusion was associated with a higher risk of outcome. Final model 4′s c-index was 0.7864, an increase of 0.0068 (95% CI 0.0044, 0.0092) compared to model 3. The Nagelkerke R2 was 0.3304. The results of the likelihood-ratio tests showed that the addition of covariates improved overall model fit at each step (Table 3). The models that included only SAPS II, only APACHE II score, and only age plus eGFR, had c-indexes of 0.6305, 0.6139, and 0.6689, respectively.

## 4. Discussion

The current study’s objective was to evaluate the added value of plasma and urinary biomarkers to conventional clinical variables and severity scores in risk stratification of ICU patients. Previous studies in ICU patients have shown that scoring systems such as the APACHE II [10,11], SAPS II [12], and SOFA [13,14], which are based on a combination of traditional clinical variables, are valid predictors of prognosis. However, these scores may add limited prognostic information over and above simple clinical and laboratory values for a variety of reasons [24,25,26,27]. Additional versions of these scoring systems (such as SAPS-III, APACHE III and IV) were developed, but their additional predictive value is small and their complexity has limited their utility [28,29]; their components were not collected in this study. Furthermore, these scores do not include newer biomarker assessments. In the current analysis, the best performing score (SAPS-II) had a lower discriminative power than a clinical model. In fact, considering only age and renal function (eGFR) at admission provided better discrimination than either the SAPS II or APACHE II score. This finding highlights the need to develop better systems to assess disease severity and gage the outcome risk of patients in the ICU. The large difference in predictive value between SAPS II and APACHE II and a clinical model suggests that these scoring systems do not capture the full clinical information available and should be developed further. 

Those patients who become more critically ill after admission to the ICU often have subsequent complications of multi-organ failure. From this point of view, the utilization, soon after admission, of biomarkers derived from a variety of sources, such as the cardiac, vascular, renal, and inflammatory systems, may provide valuable predictive information. Our study was unique in that it evaluated a large number of biomarkers and aimed to explore the potential benefits of incorporating these novel biomarkers into prognostic models and identify the most promising candidates. In this analysis, the addition of biomarkers to the clinical + scores model improved the c-index compared to the clinical + scores model alone in predicting short- and long-term mortality. Recently, numerous novel biomarkers have been explored in the context of ICUs. These biomarkers encompass bio-ADM, galectin-3, IL-6, PCT, and sST2. Bio-ADM contributes to vasodilation [30], angiogenesis induction [31], and oxidative stress protection1 [32]. Galectin-3 participates in inflammation [33], fibrosis [34], and neoplastic transformation [35], establishing it as a marker of heart failure [36]. Both IL-6, a pro-inflammatory and anti-inflammatory cytokine, and PCT, a calcitonin precursor, function as markers of inflammation and infection [37]. sST2 is involved in inflammation [38], fibrosis [39], and cardiac stress [40], making it another marker of myocyte stress. In particular, inflammatory biomarkers and especially IL-6 and PCT, as well as markers of congestion (sST2), were independently associated with mortality whether short- or long-term. These biomarkers both reflect inflammation [41,42,43,44]. Notably, the widely used biomarkers NT-proBNP and CRP did not contribute to mortality prediction in either the short- or long-term. Importantly, the discriminative power step up was most pronounced when adding biomarkers to routine clinical variables and scores. In simple terms, a perfect model that predicts a certain outcome with absolute certainty would have a c-index of 1.0 while a model that has no predictive value will have a c-index of 0.5 (the chance of flipping a coin). The c-index for in-hospital mortality for SAPS -II alone was about 0.64, and for clinical variables without or with scores it was 0.80; the addition of novel blood and urine biomarkers improved the c-index to 0.83. Complete knowledge including clinical variables, biomarkers, and early treatment brought the c-index to 0.84. 

The urinary proteome analyses have been used to identify diagnostic and prognostic markers in patients with various diseases [18]. In the present study, HF1 proteomic classifier, comprising 85 urinary peptides and reported to be a predictor of potential diastolic left ventricular dysfunction [19,20,21], was associated with an independent risk of short- and long-term adverse outcomes in ICU patients. CKD273, including 273 urinary peptides, is significantly associated with kidney fibrosis [45] and with progression to microalbuminuria and increased risk of impaired renal function [22], and was shown to be independently associated with short-term (in-ICU and in-hospital) mortality [22]. In the current analysis, these urinary biomarkers added significant prognostic information.

## 5. Limitations

The present study has several limitations. The results are based on data collected during a certain time period. Further validation in other data bases may be important. Additionally, the results require further tuning into risk scores. Newer scores such as APACHE IV and SAPS III could not be calculated because some needed variables were not collected. Finally, although most biomarkers assessed can be measured in larger laboratories, not all (such as IL-6 or sST2) are available in daily clinical practice. However, the purpose of this study is not to recommend measuring all biomarkers in every case, but to explore the potential benefits of incorporating these novel biomarkers into a prognostic model, to identify the most promising candidates that require further investigation and validation, and, eventually, to evaluate their clinical utility and potential impact on patient management and outcomes.

## 6. Conclusions

The current analysis suggests that commonly used scores for assessing risk for patients admitted to an ICU are not optimal for risk stratification. New scores should be developed including clinical, laboratory values, and biomarkers to improve our ability to risk-stratify patients admitted to ICU.

## Figures and Tables

**Table 1:** Final multivariable logistic regression model (model 4) for in-ICU mortality.

	Covariates in Final Model								Model Performance Measures *		Statistical Comparisons with Preceding Nested Model †	
	Label	Effect Size for Unit Change of: ‡	Transform	OR	Lower CI	Upper CI	*p*-Value	df	Nagelkerke R^2^	C-Index	Difference AUC (95% CI)	*p*-Value Difference LR
Model 1 Covars	Age (year)	5 years		1.12	1.06	1.18	<0.001	1				
	Male gender	Yes vs. No		1.51	1.13	2.02	0.006	1				
	Expired Volume (mL) §	10 mL		0.98	0.97	1.00	0.01	1				
	Diastolic BP (mmHg)	70 vs. 53	spline @ 70	0.66	0.51	0.87	0.01	2				
	Diuresis of 24 h	doubling	log2	0.87	0.79	0.95	0.002	1				
	Fraction of inspired oxygen (%)	50 vs. 30	spline @ 32	0.96	0.81	1.13	<0.001	2				
	Hemoglobin (g/dL)	1 g/dL		0.93	0.86	1.00	0.05	1				
	Heart Rate (bpm)	5 bpm		1.04	1.01	1.08	0.02	1				
	Lactate (mmol/L)	1.9 vs. 0.984	quadratic polynomial	1.25	1.06	1.48	0.008	2				
	PaO2/FiO2 Ratio	25		0.96	0.93	0.99	0.003	1				
	PEEP (cmH2O)	1 cmH2O		1.04	0.99	1.10	0.13	1				
	Temperature (Celsius)	1 degree Celsius		0.85	0.75	0.97	0.01	1				
	Urea (mmol/L)	1 mmol/L		1.07	0.91	1.26	0.40	1				
	White blood cell count	doubling	spline @ log2(10500)	0.75	0.63	0.90	<0.001	2				
	Diagnosis at admission: Cardiac disease	vs. Other		1.36	0.83	2.25	<0.001	1				
	Diagnosis at admission: Acute neurological disorder	vs. Other		1.87	1.07	3.27		1				
	Diagnosis at admission: Acute respiratory failure	vs. Other		1.30	0.83	2.03		1				
	Diagnosis at admission: Sepsis	vs. Other		0.94	0.63	1.40		1				
	Diagnosis at admission: Trauma	vs. Other		0.40	0.22	0.73		1				
	CV Co-morbidities: Diabetes mellitus	Yes vs. No		0.68	0.49	0.94	0.02	1				
	Non-CV Co-morbidities: Active recent malignant tumors	Yes vs. No		1.78	1.26	2.49	<0.001	1				
	Non-CV Co-morbidities: Chronic liver disease	Yes vs. No		1.65	1.06	2.56	0.03	1				
	Non-CV Co-morbidities: COPD	Yes vs. No		1.77	1.24	2.52	0.002	1				
	Non-CV Co-morbidities: Smoking	Yes vs. No		0.69	0.50	0.94	0.02	1	0.3110	0.8084 (0.8036, 0.8133)	N/A	N/A
Model 2 Add Covars	SAPS II	2		1.02	1.00	1.03	0.02	1	0.3165	0.8117 (0.8067, 0.8167)	0.0033 (0.0023, 0.0043)	0.003
Model 3 Add Covars	IL-6	doubling	log2	1.17	1.09	1.26	<0.001	1				
	PCT	doubling	spline @ log2(1.9)	0.98	0.87	1.10	<0.001	2				
	Soluble-ST2	doubling	log2	1.20	1.04	1.39	0.01	1				
	Proteomic Classifier: HF1	0.1		1.08	1.01	1.15	0.01	1				
	Proteomic Classifier: CKD273	0.1		1.03	1.01	1.05	<0.001	1	0.3564	0.8269 (0.8220, 0.8318)	0.0152 (0.0163, 0.0207)	<0.001
Model 4 Add Covars	Cardiac arrest before admission	Yes vs. No		2.44	1.44	4.16	0.001	1				
	Chronic Treatment: Aldosterone antagonists	Yes vs. No		4.93	1.51	16.03	0.008	1				
	Meds from admission to inclusion: Feeding Enteral	Yes vs. No		1.37	1.04	1.80	0.03	1				
	Meds from admission to inclusion: Feeding Parenteral	Yes vs. No		1.74	1.26	2.41	<0.001	1	0.3707	0.8388 (0.8334, 0.8442)	0.0119 (0.0095, 0.0142)	<0.001

* Performance measures are for the model including only those covariates in the specific model, in which parameter estimates may differ from those shown for final model 4. † Statistics are shown comparing the model including the added covariates plus all preceding covariates with the model including only the preceding covariates. ‡ Effect presented for 75th vs. 25th percentiles for transformed variables (spline, quadratic, cubic). § Expired volume is the volume measured by the ventilator at expiration.

**Table 2:** Final multivariable logistic regression model (model 4) for in-hospital mortality.

	Covariates in Final Model								Model Performance Measures *		Statistical Comparisons with Preceding Nested Model †	
	Label	Effect Size for Unit Change of: ‡	Transform	OR	Lower CI	Upper CI	*p*-Value	df	Nagelkerke R^2^	C-Index	Difference AUC (95% CI)	p-Value Difference LR
Model 1 Covars	Age (year)	5 years		1.16	1.11	1.22	<0.001	1				
	Male gender	Yes vs. No		1.61	1.22	2.12	<0.001	1				
	Diastolic BP (mmHg)	70 vs. 53	spline @ 70	0.69	0.54	0.88	0.01	2				
	Diuresis of 24 h	doubling	log2	0.88	0.81	0.96	0.003	1				
	Expired Volume (mL) §	10 mL		0.98	0.97	1.00	0.01	1				
	Fraction of inspired oxygen (%)	50 vs. 30	cubic polynomial	0.72	0.55	0.94	0.001	3				
	Hemoglobin (g/dL)	1 g/dL		0.94	0.88	1.01	0.11	1				
	Heart Rate (bpm)	5 bpm		1.01	0.98	1.04	0.42	1				
	Lactate (mmol/L)	1.9 vs. 0.984	quadratic polynomial	1.16	0.99	1.37	0.12	2				
	PaO2/FiO2 Ratio	25		0.96	0.93	0.99	0.003	1				
	Temperature (Celsius)	1 degree Celsius		0.89	0.79	1.01	0.07	1				
	Urea	3.8074 vs. 2.3998	cubic polynomial	1.15	0.87	1.54	0.76	3				
	White blood cell count	doubling	spline @ log2(10500)	0.69	0.58	0.81	<0.001	2				
	Diagnosis at admission: Cardiac disease	vs. Other		1.21	0.76	1.93	<0.001	1				
	Diagnosis at admission: Acute neurological disorder	vs. Other		1.66	1.00	2.74		1				
	Diagnosis at admission: Acute respiratory failure	vs. Other		1.20	0.80	1.81		1				
	Diagnosis at admission: Sepsis	vs. Other		0.84	0.58	1.21		1				
	Diagnosis at admission: Trauma	vs. Other		0.42	0.25	0.73		1				
	Non-CV Co-morbidities: Active recent malignant tumors	Yes vs. No		1.62	1.18	2.22	0.003	1				
	Non-CV Co-morbidities: Chronic liver disease	Yes vs. No		1.78	1.17	2.70	0.007	1				
	Non-CV Co-morbidities: COPD	Yes vs. No		1.51	1.07	2.11	0.02	1				
	Non-CV Co-morbidities: Loss of autonomy	Yes vs. No		1.95	1.13	3.36	0.02	1				
	Non-CV Co-morbidities: Smoking (active or stopped past year)	Yes vs. No		0.74	0.56	0.99	0.04	1	0.3097	0.8021 (0.7975, 0.8066)	N/A	N/A
Model 2 Add Covars	SAPS II	2		1.01	1.00	1.02	0.17	1	0.3132	0.8039 (0.7994, 0.8083)	0.0018 (0.0013, 0.0024)	0.02
Model 3 Add Covars	Bioactive-adrenomedullin	doubling	log2	1.13	1.00	1.29	0.05	1				
	Galectin-3	doubling	log2	1.32	1.09	1.59	0.004	1				
	IL-6	doubling	log2	1.13	1.06	1.21	<0.001	1				
	PCT	doubling	spline @ log2(1.9)	0.98	0.89	1.09	<0.001	2				
	Soluble-ST2	doubling	log2	1.24	1.08	1.41	0.002	1				
	Proteomic Classifier: HF1	0.1		1.03	1.01	1.05	<0.001	1	0.3605	0.8269 (0.8227, 0.8311)	0.0230 (0.0214, 0.0246)	<0.001
Model 4 Add Covars	Cardiac arrest before admission	Yes vs. No		2.38	1.44	3.94	<0.001	1				
	Chronic Treatment: Antidiabetics	Yes vs. No		0.61	0.42	0.89	0.01	1				
	Meds from admission to inclusion: Morphine	Yes vs. No		0.70	0.54	0.91	0.009	1				
	Meds from admission to inclusion: Feeding Parenteral	Yes vs. No		1.43	1.06	1.92	0.02	1	0.3651	0.8356 (0.8307, 0.8405)	0.0087 (0.0067, 0.0107)	<0.001

* Performance measures are for the model including only those covariates in the specific model, in which parameter estimates may differ from those shown for final model 4. † Statistics are shown comparing the model including the added covariates plus all preceding covariates with the model including only the preceding covariates. ‡ Effect presented for 75th vs. 25th percentiles for transformed variables (spline, quadratic, cubic). § Expired volume is the volume measured by the ventilator at expiration.

**Table 3:** Final multivariable Cox regression model (model 4) for 1 year mortality.

	Covariates in Final Model								Model Performance Measures *		Statistical Comparisons with Preceding Nested Model †	
	Label	Effect Size for Unit Change of: ‡	Transform	HR	Lower CI	Upper CI	*p*-Value	df	Nagelkerke R^2^	C-Index	Difference C-Index (95% CI)	*p*-Value Difference LR
Model 1 Covars	Age (year)	5 years		1.10	1.05	1.14	<0.001	1				
	Male gender	Yes vs. No		1.38	1.17	1.63	<0.001	1				
	Bicarbonates (mmol/L)	26 vs. 21	spline @ 27	0.90	0.79	1.02	0.02	2				
	Diastolic BP (mmHg)	70 vs. 53	spline @ 75	0.82	0.71	0.95	0.02	2				
	Diuresis of 24 h	doubling	log2	0.91	0.87	0.96	<0.001	1				
	Fraction of inspired oxygen (%)	50 vs. 30	spline @ 45	0.71	0.58	0.86	<0.001	2				
	Hemoglobin (g/dL)	1 g/dL		0.96	0.92	1.00	0.06	1				
	Heart Rate (bpm)	5 bpm		1.01	0.99	1.04	0.18	1				
	Lactate (mmol/L)	1.9 vs. 0.984	quadratic polynomial	1.06	0.97	1.17	0.007	2				
	PaO2/FiO2 Ratio	25		0.97	0.96	0.99	0.003	1				
	PEEP (cmH2O)	1 cmH2O		1.04	1.00	1.07	0.04	1				
	Temperature (Celsius)	1 degree Celsius	quadratic polynomial	0.90	0.81	0.99	0.03	2				
	Urea (mmol/L)	1 mmol/L		0.99	0.90	1.09	0.80	1				
	Weight (Kg)	5 Kg		0.96	0.93	0.98	<0.001	1				
	White blood cell count	doubling	spline @ log2(10900)	0.81	0.73	0.90	<0.001	2				
	Diagnosis at admission: Cardiac disease	vs. Other		1.49	1.11	2.01	<0.001	1				
	Diagnosis at admission: Acute neurological disorder	vs. Other		1.39	1.00	1.94		1				
	Diagnosis at admission: Acute respiratory failure	vs. Other		1.38	1.06	1.78		1				
	Diagnosis at admission: Sepsis	vs. Other		1.13	0.90	1.42		1				
	Diagnosis at admission: Trauma	vs. Other		0.67	0.48	0.95		1				
	Oxygen at home	Yes vs. No		1.73	1.04	2.88	0.03	1				
	Non-CV Co-morbidities: Active recent malignant tumors	Yes vs. No		1.46	1.19	1.80	<0.001	1				
	Non-CV Co-morbidities: Chronic liver disease	Yes vs. No		1.17	0.87	1.59	0.30	1				
	Non-CV Co-morbidities: Loss of autonomy	Yes vs. No		1.87	1.38	2.55	<0.001	1	0.2584	0.7557 (0.7519, 0.7596)	N/A	N/A
Model 2 Add Covars	SAPS II	2		1.01	1.00	1.02	0.002	1				
	Charlson Comorbidity Index	doubling	log2	1.18	1.02	1.37	0.03	1	0.2617	0.7602 (0.7564, 0.7639)	0.0044 (0.0029, 0.0059)	<0.001
Model 3 Add Covars	Bioactive-adrenomedullin	doubling	log2	1.09	1.00	1.18	0.04	1				
	Galectin-3	doubling	log2	1.21	1.07	1.36	0.002	1				
	IL-6	doubling	log2	1.13	1.08	1.18	<0.001	1				
	PCT	doubling	spline @ log2(1.9)	0.92	0.86	0.99	<0.001	2				
	Soluble-ST2	doubling	log2	1.14	1.04	1.24	0.004	1				
	Proteomic Classifier: HF1	0.1		1.02	1.01	1.03	0.004	1				
	Proteomic Classifier: ACM128	0.1		1.03	1.01	1.04	<0.001	1	0.3187	0.7796 (0.7767, 0.7826)	0.0195 (0.0168, 0.0222)	<0.001
Model 4 Add Covars	Cardiac arrest before admission	Yes vs. No		1.39	1.01	1.93	0.05	1				
	Chronic Treatment: Aldosterone antagonists	Yes vs. No		2.29	1.18	4.43	0.01	1				
	Chronic Treatment: Morphine	Yes vs. No		1.60	1.10	2.35	0.01	1				
	Chronic Treatment: Nitrates	Yes vs. No		0.31	0.13	0.78	0.01	1				
	Meds from admission to inclusion: Morphine	Yes vs. No		0.84	0.71	0.99	0.04	1				
	Meds from admission to inclusion: Renal replacement therapy	Yes vs. No		0.74	0.58	0.94	0.01	1				
	Meds from admission to inclusion: Feeding Parenteral	Yes vs. No		1.27	1.05	1.53	0.01	1	0.3304	0.7864 (0.7830, 0.7899)	0.0068 (0.0044, 0.0092)	<0.001

* Performance measures are for the model including only those covariates in the specific model, in which parameter estimates may differ from those shown for final model 4. † Statistics are shown comparing the model including the added covariates plus all preceding covariates with the model including only the preceding covariates. ‡ Effect presented for 75th vs. 25th percentiles for transformed variables (spline, quadratic, cubic).

## Data Availability

A.M. had full access to all the study data and takes responsibility for the integrity of the data and the accuracy of the data analysis.

## Supplementary Materials

The following supporting information can be downloaded at: https://www.mdpi.com/article/10.3390/jcm12093311/s1, Table S1a: Continuous covariates considered for inclusion in the prognostic models; Table S1b: Categorical variables considered for inclusion in the prognostic models; Table S2: Centrally measured biomarkers at study inclusion considered in prognostic models; Figure S1: Calibration Plots for Multivariable Logistic Regression and Cox Proportional Hazards Models for (A) In-ICU Mortality, (B) In-Hospital Mortality, and (C) 1 Year Survival; Figure S2: Receiver Operating Characteristic Curves for Multivariable Logistic Regression Models for (A) In-ICU Mortality, (B) In-Hospital Mortality, and (C) 1-Year Survival.

## Abbreviations

APACHE	Acute Physiology and Chronic Health Evaluation
bio-ADM	Bio-active adrenomedullin
CI	Confidence interval
eGFR	Estimated glomerular filtration rate
FROG-ICU	French and European Outcome reGistry in Intensive Care Units
HR	Hazard ratio
ICU	Intensive care unit
IL-6	Interleukin 6
NT-proBNP	N-terminal pro-B type natriuretic peptide
OR	Odds ratio
PCT	Procalcitonin
ROC	Receiver operating characteristic
SAPS	Simplified Acute Physiology Score
SOFA	Sequential Organ Failure Assessment
sST2	Soluble-ST2
