# Supplementary material for: Plasma and Urinary Biomarkers Improve Prediction of Mortality through 1 Year in Intensive Care Patients: An Analysis from FROG-ICU
Source: J Clin Med. 2023 May 6;12(9):3311. doi: 10.3390/jcm12093311 (PMC10179283; doi:10.3390/jcm12093311)

**Supplementary material**

# Plasma and Urinary Biomarkers Improve Prediction of Mortality through 1 Year in Intensive Care Patients: An Analysis from FROG-ICU

Beth A. Davison, Christopher Edwards, Gad Cotter, Antoine Kimmoun, Étienne Gayat, Agnieszka Latosinska, Harald Mischak, Koji Takagi, Benjamin Deniau, Adrien Picod, Alexandre Mebazaa

Table of Contents

Table S1a. Continuous covariates considered for inclusion in the prognostic models. ................................ 2

Table S1b. Categorical variables considered for inclusion in the prognostic models.................................. 4

Table S2. Centrally measured biomarkers at study inclusion considered in prognostic models. ................. 7

Figure S1. Calibration Plots for Multivariable Logistic Regression and Cox Proportional Hazards Models for A) In-ICU Mortality, B) In-Hospital Mortality, and C) 1-Year Survival............................................... 8

Figure S2. Receiver Operating Characteristic Curves for Multivariable Logistic Regression Models for A) In-ICU Mortality, B) In-Hospital Mortality, and C) 1-Year Survival ........................................................ 9

**Table S1a. Continuous covariates considered for inclusion in the prognostic models.**

| | N | Missing (%) | Mean | SD | Min | Q1 | Median | Q3 | Max |
|---|---|---|---|---|---|---|---|---|---|
| **Demographics** | | | | | | | | | |
| Age (year) | 2087 | 0.0 | 61 | 16 | 18 | 51 | 63 | 74 | 98 |
| **Vitals at Inclusion** | | | | | | | | | |
| Systolic BP (mmHg) | 2037 | 2.4 | 124 | 23 | 40 | 108 | 122 | 139 | 220 |
| Diastolic BP (mmHg) | 1967 | 5.7 | 63 | 14 | 10 | 53 | 61 | 70 | 149 |
| Heart Rate (bpm) | 2009 | 3.7 | 93 | 21 | 42 | 78 | 92 | 106 | 190 |
| Respiratory Rate (/min) | 1546 | 25.9 | 21 | 7 | 8 | 16 | 20 | 25 | 51 |
| Temperature (Celsius) | 2040 | 2.3 | 37.2 | 1.0 | 27.0 | 36.7 | 37.2 | 37.8 | 40.9 |
| Oxygen Saturation (%) | 1976 | 5.3 | 97 | 3 | 70 | 96 | 98 | 100 | 100 |
| Weight (Kg) | 1750 | 16.1 | 79 | 19 | 30 | 65 | 78 | 90 | 212 |
| Positive End-Expiratory Pressure (cmH2O) | 1843 | 11.7 | 6 | 3 | 1 | 5 | 5 | 8 | 28 |
| Expired volume (ml) | 1558 | 25.3 | 484 | 118 | 60 | 410 | 470 | 534 | 1000 |
| Fraction of inspired oxygen (%) | 1920 | 8.0 | 43 | 17 | 21 | 30 | 40 | 50 | 100 |
| PaO2/FiO2 Ratio | 1770 | 15.2 | 224.8 | 140.9 | 2.0 | 123.3 | 220.0 | 316.7 | 709.5 |
| Partial pressure of carbon dioxide (mmHg) | 1894 | 9.2 | 39 | 9 | 20 | 34 | 38 | 43 | 126 |
| Partial pressure of oxygen (mmHg) | 1912 | 8.4 | 106 | 53 | 30 | 76 | 92 | 120 | 600 |
| **Local Laboratory at Inclusion** | | | | | | | | | |
| White blood cell count (/mm3) | 1938 | 7.1 | 12374 | 7496 | 500 | 7600 | 10900 | 16100 | 65100 |
| Hemoglobin (g/dL) | 1983 | 5.0 | 10.2 | 1.9 | 3.4 | 8.9 | 10.0 | 11.4 | 19.9 |
| Platelets (/mm3) | 1958 | 6.2 | 185750 | 120760 | 3000 | 99000 | 164000 | 243000 | 860000 |
| Total protein (g/L) | 1835 | 12.1 | 54 | 10 | 20 | 48 | 54 | 60 | 112 |
| Urea (mmol/L) | 1936 | 7.2 | 10.7 | 8.3 | 0.3 | 5.3 | 8.4 | 14.0 | 78.3 |
| eGFR (ml/min/1.73 m2) | 2087 | 0.0 | 80.4 | 44.9 | 6.0 | 40.0 | 77.5 | 117.6 | 150.0 |
| Uric acid (µmol/L) | 1984 | 4.9 | 217.0 | 139.4 | 30.0 | 116.5 | 183.0 | 288.6 | 1175.8 |
| Sodium (mmol/L) | 2030 | 2.7 | 140 | 5 | 116 | 137 | 140 | 143 | 158 |
| Potassium (mmol/L) | 2008 | 3.8 | 3.9 | 0.6 | 2.6 | 3.6 | 3.9 | 4.3 | 7.0 |
| Chloride (mmol/L) | 2077 | 0.5 | 102 | 7 | 70 | 99 | 103 | 107 | 129 |
| Glycemia (mmol/L) | 1958 | 6.2 | 7.9 | 2.7 | 0.1 | 6.2 | 7.4 | 9.1 | 26.9 |
| Lactate (mmol/L) | 1647 | 21.1 | 1.71 | 1.51 | 0.06 | 0.98 | 1.30 | 1.90 | 16.00 |
| Arterial pH | 1905 | 8.7 | 7.41 | 0.08 | 7.00 | 7.36 | 7.42 | 7.46 | 7.84 |
| Bicarbonates (mmol/L) | 1758 | 15.8 | 24 | 5 | 6 | 21 | 24 | 26 | 50 |
| Diuresis of 24 hours (ml/24h) | 1690 | 19.0 | 1652 | 1254 | 1 | 800 | 1388 | 2200 | 10000 |
| **Central Laboratory/Biomarkers at Inclusion** | | | | | | | | | |

| | | | | | | | | |
|---|---|---|---|---|---|---|---|---|
| Soluble-ST2 (ng/mL) | 2063 | 1.1 | 520.0 | 455.5 | 3.5 | 182.3 | 345.6 | 765.1 | 2321.1 |
| Proenkephalin (pmol/L) | 2075 | 0.6 | 100.4 | 116.1 | 11.5 | 36.5 | 61.2 | 116.2 | 1398.6 |
| Bioactive-adrenomedullin (pg/mL) | 2074 | 0.6 | 116.1 | 156.1 | 4.0 | 34.7 | 66.6 | 135.2 | 1926.1 |
| Galectin-3 (ng/mL) | 2076 | 0.5 | 27.4 | 20.1 | 5.2 | 14.2 | 21.0 | 33.0 | 114.0 |
| PCT (ng/mL) | 2075 | 0.6 | 8.3 | 20.9 | 0.0 | 0.3 | 1.1 | 5.8 | 186.7 |
| IL-6 (pg/mL) | 2076 | 0.5 | 678.2 | 3622.4 | 1.5 | 43.5 | 100.9 | 261.7 | 50000.0 |
| CRP (mg/L) | 2077 | 0.5 | 159.1 | 102.2 | 0.6 | 78.6 | 143.7 | 219.8 | 547.3 |
| NT-proBNP (pg/mL) | 2076 | 0.5 | 4828 | 8486 | 5 | 293 | 1202 | 4516 | 35000 |
| Troponin T (pg/mL) | 2076 | 0.5 | 306.6 | 1001.6 | 3.0 | 17.5 | 45.7 | 143.1 | 10000.0 |
| Cystatin-C (mg/L) | 2077 | 0.5 | 1.7 | 1.1 | 0.0 | 0.9 | 1.3 | 2.1 | 9.5 |
| NGAL (ng/mL) | 2041 | 2.2 | 428 | 613 | 1 | 97 | 209 | 509 | 7668 |
| Urinary Albumin (mg/L) | 2077 | 0.5 | 1088 | 1473 | 84 | 300 | 384 | 478 | 4000 |
| Urinary Cystatin-C (mg/L) | 1926 | 7.7 | 2.6 | 5.7 | 0.0 | 0.1 | 0.3 | 1.9 | 50.1 |
| Urinary NGAL (ng/mL) | 1927 | 7.7 | 454.9 | 564.5 | 0.9 | 38.8 | 135.4 | 740.8 | 1500.0 |
| Urinary L-FABP (ng/mL) | 1916 | 8.2 | 450.4 | 3160.2 | 0.1 | 10.3 | 31.6 | 92.0 | 64800.0 |
| **Disease Severity Scores at Admission** | | | | | | | | | |
| APACHE II | 971 | 53.5 | 22.29 | 7.67 | 3 | 17 | 23 | 28 | 42 |
| SOFA score | 1518 | 27.3 | 8 | 4 | 0 | 5 | 8 | 10 | 22 |
| SAPS II | 2086 | 0.0 | 50 | 19 | 0 | 36 | 49 | 63 | 110 |
| Glasgow Coma Scale | 1696 | 18.7 | 11 | 5 | 3 | 5 | 14 | 15 | 15 |
| Charlson Comorbidity Index | 1736 | 16.8 | 4 | 2 | 1 | 2 | 4 | 5 | 12 |
| **Novel Urinary Biomarker at Inclusion** | | | | | | | | | |
| HF1-classifier | 1649 | 21.0 | -0.09 | 0.95 | -3.38 | -0.75 | -0.09 | 0.64 | 2.53 |
| HF2-classifier | 1649 | 21.0 | -0.06 | 0.59 | -2.21 | -0.45 | -0.06 | 0.33 | 2.22 |
| CAD238-classifier | 1649 | 21.0 | -0.34 | 0.26 | -1.06 | -0.51 | -0.39 | -0.21 | 0.95 |
| CKD273-classifier | 1686 | 19.2 | 0.44 | 0.39 | -1.11 | 0.21 | 0.56 | 0.73 | 1.19 |
| ACM128-classifier | 1624 | 22.2 | -0.27 | 0.69 | -1.98 | -0.76 | -0.35 | 0.16 | 2.39 |

**Table S1b. Categorical variables considered for inclusion in the prognostic models.**

| | Missing (%) | n | N | % |
|---|---|---|---|---|
| **Demographics** | | | | |
| Male gender | 0 | 1361 | 2087 | 65.2 |
| **Diagnosis at Admission** | 0 | | | |
| Cardiac disease | | 325 | 2086 | 15.6 |
| Acute neurological disorder | | 286 | 2086 | 13.7 |
| Acute respiratory failure | | 394 | 2086 | 18.9 |
| Sepsis | | 536 | 2086 | 25.7 |
| Trauma | | 199 | 2086 | 9.5 |
| Other | | 346 | 2086 | 16.6 |
| **Admission Unit** | 0 | | | |
| Cardiac Ward | | 91 | 2087 | 4.4 |
| Emergency Room | | 563 | 2087 | 27 |
| Home | | 206 | 2087 | 9.9 |
| Medical | | 9 | 2087 | 0.4 |
| Medical ICU | | 95 | 2087 | 4.6 |
| Medical Ward | | 274 | 2087 | 13.1 |
| Operating Room | | 187 | 2087 | 9 |
| Scheduled Surgical | | 51 | 2087 | 2.4 |
| Surgical ICU | | 168 | 2087 | 8 |
| Surgical Ward | | 219 | 2087 | 10.5 |
| Unscheduled Surgical | | 47 | 2087 | 2.3 |
| Trauma | | 47 | 2087 | 2.3 |
| Other | | 130 | 2087 | 6.2 |
| **Status at Admission** | | | | |
| Cardiac arrest before admission | 0.2 | 178 | 2082 | 8.5 |
| Kidney Disease Improvement Global Outcome | 4.3 | | | |
| 0 | | 1485 | 1998 | 74.3 |
| 1 | | 309 | 1998 | 15.5 |
| 2 | | 133 | 1998 | 6.7 |
| 3 | | 71 | 1998 | 3.6 |
| **Chronic treatments** | | | | |
| Oxygen at home | 0.9 | 29 | 2068 | 1.4 |
| Aldosterone antagonists | 0.9 | 14 | 2068 | 0.7 |
| Diuretics | 0.9 | 449 | 2068 | 21.7 |
| Antiplatelets | 0.9 | 533 | 2068 | 25.8 |
| Calcium antagonist | 0.9 | 285 | 2068 | 13.8 |
| Inhaled steroids | 0.9 | 82 | 2068 | 4 |
| Psychiatric treatment | 0.9 | 457 | 2068 | 22.1 |
| Morphine | 0.9 | 53 | 2068 | 2.6 |
| Insulin | 0.9 | 118 | 2068 | 5.7 |

| | | | | |
|---|---|---|---|---|
| ACE Inhibitors or ARB | 0.9 | 553 | 2068 | 26.7 |
| Cardiac glycosides | 0.9 | 30 | 2068 | 1.5 |
| Nitrates | 0.9 | 15 | 2068 | 0.7 |
| Vitamin K Antagonists | 0.9 | 173 | 2068 | 8.4 |
| Statins | 0.9 | 497 | 2068 | 24 |
| Beta-2 mimetics | 0.9 | 104 | 2068 | 5 |
| Antidiabetics | 0.9 | 222 | 2068 | 10.7 |
| Beta Blocker Non cardio selective | 0.9 | 155 | 2068 | 7.5 |
| Beta Blocker Cardio selective | 0.9 | 370 | 2068 | 17.9 |
| Amiodarone | 0.9 | 109 | 2068 | 5.3 |
| Immunosuppressive agents | 0.9 | 60 | 2068 | 2.9 |
| Hydrocortisone | 0.9 | 163 | 2068 | 7.9 |
| **CV Comorbidities** | | | | |
| Chronic heart failure | 0.2 | 153 | 2083 | 7.3 |
| Diabetes mellitus | 0.2 | 384 | 2083 | 18.4 |
| Hypertension | 0.2 | 902 | 2083 | 43.3 |
| Prior myocardial infarction | 0.2 | 83 | 2083 | 4 |
| Severe valvular disease or previous valvular surgery | 0.2 | 82 | 2083 | 3.9 |
| Prior stroke | 0.2 | 92 | 2083 | 4.4 |
| Peripheral vascular disease including carotid artery disease | 0.2 | 209 | 2083 | 10 |
| Pulmonary embolism | 0.2 | 58 | 2083 | 2.8 |
| Dyslipidemia | 0.2 | 412 | 2083 | 19.8 |
| Obesity | 0.2 | 227 | 2083 | 10.9 |
| Coronary artery disease | 0.2 | 188 | 2083 | 9 |
| Atrial fibrillation flutter | 0.2 | 221 | 2083 | 10.6 |
| Pulmonary hypertension | 0.2 | 33 | 2083 | 1.6 |
| Congenital heart defect | 0.2 | 11 | 2083 | 0.5 |
| Pacemaker | 0.2 | 54 | 2083 | 2.6 |
| Cardiac resynchronization therapy | 0.2 | 11 | 2083 | 0.5 |
| Prior Coronary Revascularization | 0.2 | 126 | 2083 | 6 |
| Cardiac Defibrillator | 0.2 | 16 | 2083 | 0.8 |
| **Non-CV Comorbidities** | | | | |
| COPD | 0.2 | 273 | 2083 | 13.1 |
| Chronic liver disease | 0.2 | 158 | 2083 | 7.6 |
| Depression | 0.2 | 261 | 2083 | 12.5 |
| Dysthyroidism | 0.2 | 140 | 2083 | 6.7 |
| Cognitive dysfunction | 0.2 | 33 | 2083 | 1.6 |
| Smoking | 0.2 | 570 | 2083 | 27.4 |
| Alcohol | 0.2 | 365 | 2083 | 17.5 |
| Hemodialysis | 0.2 | 28 | 2083 | 1.3 |
| Chronic renal disease | 0.2 | 241 | 2083 | 11.6 |
| Active recent malignant tumors | 0.2 | 281 | 2083 | 13.5 |
| Asthma | 0.2 | 93 | 2083 | 4.5 |

| | | | | |
|---|---|---|---|---|
| Anemia | 0.2 | 33 | 2083 | 1.6 |
| Chronic inflammatory disease | 0.2 | 77 | 2083 | 3.7 |
| Loss of autonomy | 0.2 | 78 | 2083 | 3.7 |
| HIV | 0.2 | 53 | 2083 | 2.5 |
| **Medications (Admission to Inclusion)** | | | | |
| Stockings compression | 0.3 | 521 | 2080 | 25 |
| Inotrope/vasopressor | 0.3 | 1490 | 2080 | 71.6 |
| RBC Transfusion | 0.3 | 476 | 2080 | 22.9 |
| Feeding Enteral | 0.3 | 1032 | 2080 | 49.6 |
| Hydrocortisone | 0.3 | 581 | 2080 | 27.9 |
| Renal Replacement Therapy | 0.3 | 219 | 2080 | 10.5 |
| ECMO | 0.3 | 28 | 2080 | 1.3 |
| Feeding Parenteral | 0.3 | 363 | 2080 | 17.5 |
| Coronary revascularization | 0.3 | 37 | 2080 | 1.8 |
| Heparin | 0.3 | 1577 | 2080 | 75.8 |
| Other device | 0.3 | 442 | 2080 | 21.2 |
| Vasodilator | 0.3 | 196 | 2080 | 9.4 |
| Morphine | 0.3 | 1539 | 2080 | 74 |
| Benzodiazepine | 0.3 | 1028 | 2080 | 49.4 |
| Neuromuscular blocking agents (curare) | 0.3 | 720 | 2080 | 34.6 |
| Amiodarone | 0.3 | 334 | 2080 | 16.1 |

ACE, angiotensin-converting enzyme; ARB, angiotensin receptor blocker; COPD, chronic obstructive pulmonary disease; ECMO, extracorporeal membrane oxygenation; HIV, human immunodeficiency virus; ICU, intensive care unit, RBC, red blood cells.

**Table S2. Centrally measured biomarkers at study inclusion considered in prognostic models.**

| Biomarker | Assay | Function and clinical implication |
|---|---|---|
| ***Plasma biomarkers included in model(s)*** | | |
| Bioactive-adrenomedullin (pg/mL) | Adrenomed GmbH, Hennigsdorf, Germany | vasodilatation, induction of angiogenesis, protection against oxidative stress and hypoxic injury: marker of myocyte stress |
| Galectin-3 (ng/mL) | Abbott, Abbott Park, IL, USA | involve in inflammation, fibrosis and neoplastic transformation: marker of heart failure |
| Interleukin-6 (pg/mL) | Roche, Penzberg, Germany | pro-inflammation and anti-inflammation cytokine: marker of inflammation/infection |
| Procalcitonin (ng/mL) | Adrenomed GmbH, Hennigsdorf, Germany | precursor of calcitonin: marker of infection, mostly bacterial |
| Soluble-ST2 (ng/mL) | Eurobio, Critical Diagnostics, San Diego, CA, USA | involve in inflammation, fibrosis, and cardiac stress: marker of myocyte stress |
| ***Plasma biomarkers also considered*** | | |
| C-reactive protein (mg/L) | Abbott, Abbott Park, IL, USA | acute-phase protein: marker of inflammation/infection |
| Cystatin-C (mg/L) | Abbott, Abbott Park, IL, USA | protein derived by all nucleated cells, reabsorbed by proximal tubular cells: marker of decrease glomerular filtration rate |
| NGAL (ng/mL) | Abbott, Abbott Park, IL, USA | involve in innate immunity: marker of renal tubular injury |
| NT-proBNP (pg/mL) | Roche Diagnostics GmbH, Mannheim, Germany | biologically inactive segment of BNP: marker of myocyte stress |
| Proenkephalin (pmol/L) | Sphingotec GmbH, Hennigsdorf, Germany | endogenous opioid polypeptide hormone: marker of cardiovascular and cerebrovascular disease |
| Troponin T (pg/mL) | Abbott, Abbott Park, IL, USA | part of troponin complex, heart contraction: marker of myocyte injury |
| ***Urinary biomarkers included in model(s)*** | | |
| Proteomic Classifier: ACM128 | Mosaiques Diagnostics, Hanover, Germany | consist of 128 urinary peptide fragments |
| Proteomic Classifier: CKD273 | Mosaiques Diagnostics, Hanover, Germany | consist of 273 urinary peptide fragments: marker of early fibrosis of the kidney |
| Proteomic Classifier: HF1 | Mosaiques Diagnostics, Hanover, Germany | consist of 85 urinary peptide fragments: marker of left ventricular dysfunction |
| ***Urinary biomarkers also considered*** | | |
| Urinary Albumin (mg/L) | Abbott, Abbott Park, IL, USA | involve in glomerular injury: marker of renal glomerular injury |
| Urinary Cystatin-C (mg/L) | Abbott, Abbott Park, IL, USA | protein derived by all nucleated cells, reabsorbed by proximal tubular cells: marker of renal tubular injury |
| Urinary L-FABP (ng/mL) | Nordia L-FABP; Sekisui Medical Co., Ltd., Tokyo, Japan | involve in renal tubulointerstitial damage: marker of renal tubular injury |
| Urinary NGAL (ng/mL) | Abbott, Abbott Park, IL, USA | involve in innate immunity: marker of renal tubular injury |
| Proteomic Classifier: CAD238 | Mosaiques Diagnostics, Hanover, Germany | consist of 238 urinary peptide fragments: marker of cardiovascular disease |
| Proteomic Classifier: HF2 | Mosaiques Diagnostics, Hanover, Germany | consists of 671 urinary peptide fragments: marker of left ventricular dysfunction |

**Figure S1. Calibration Plots for Multivariable Logistic Regression and Cox Proportional Hazards Models for A) In-ICU Mortality, B) In-Hospital Mortality, and C) 1-Year Survival**. Results are presented for the first multiple imputation dataset.

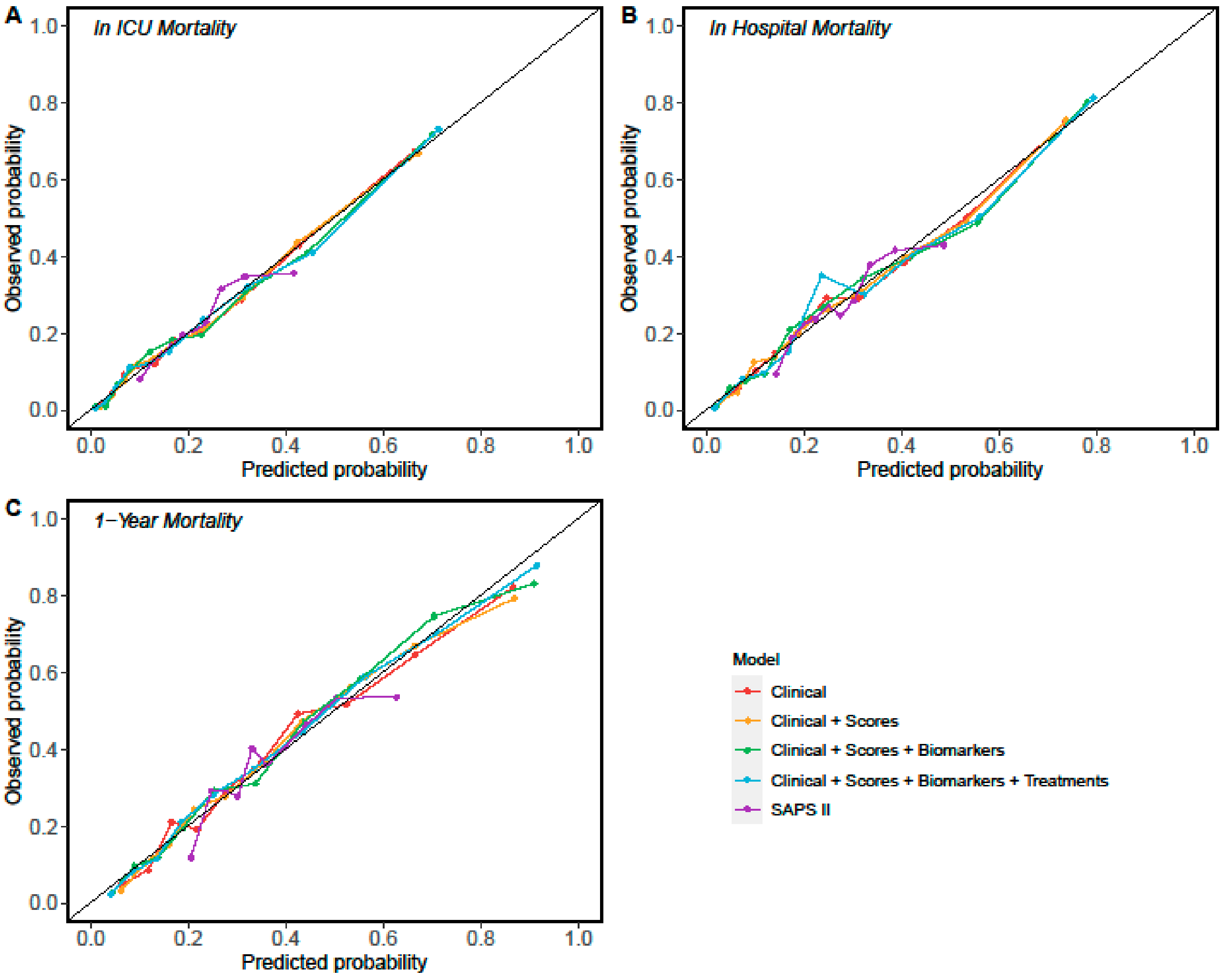

**Figure S2. Receiver Operating Characteristic Curves for Multivariable Logistic Regression Models for A) In-ICU Mortality, B) In-Hospital Mortality, and C) 1-Year Survival**. Results are presented for the first multiple imputation dataset.

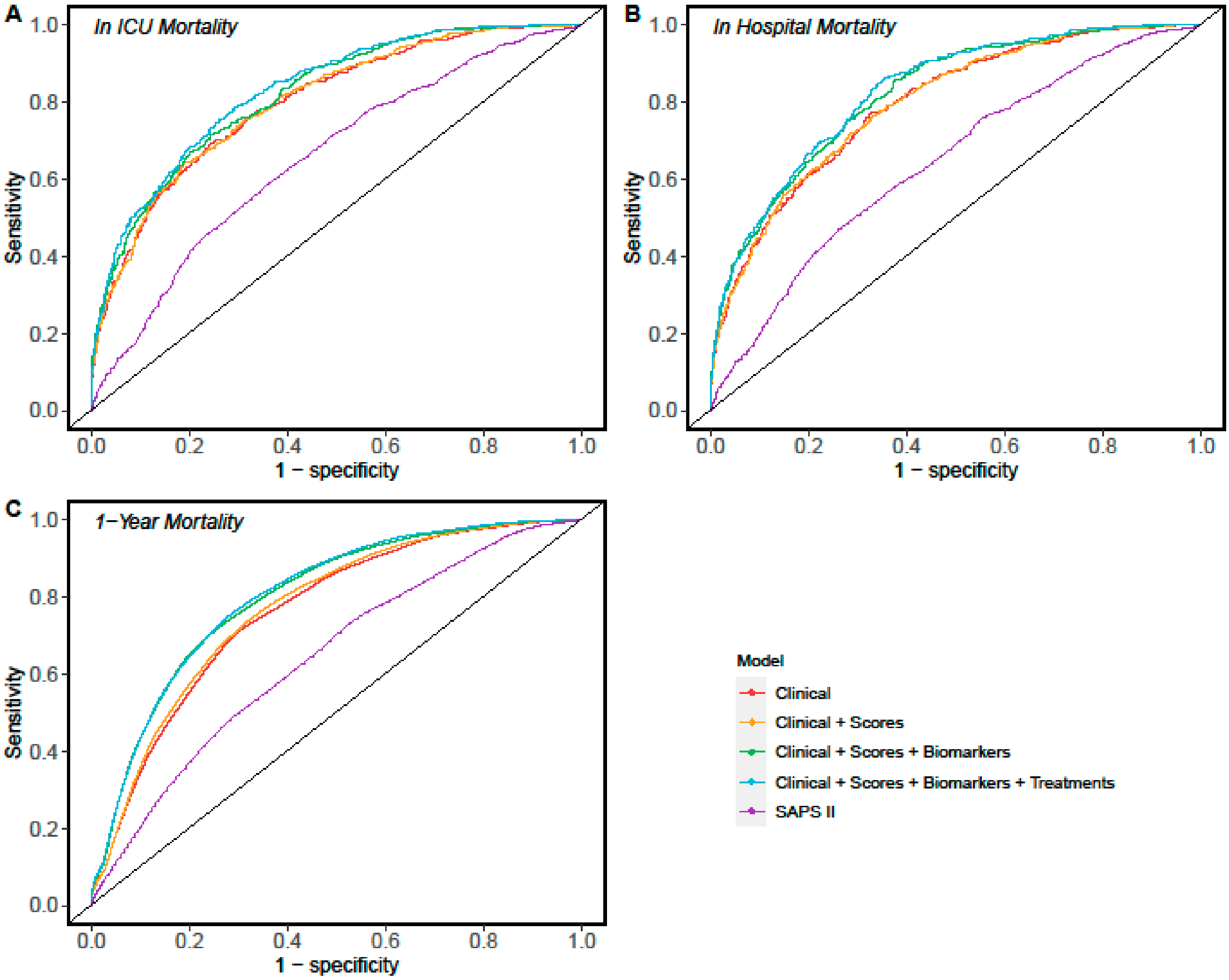

Supplement: Supplementary file 1 [file jcm-12-03311-s001.zip › jcm-2373276-supplementary.pdf]
